# Immune Alterations Following Neurological Disorders: A Comparison of Stroke and Seizures

**DOI:** 10.3389/fneur.2020.00425

**Published:** 2020-06-02

**Authors:** Johanna Ruhnau, Johanna Tennigkeit, Sonya Ceesay, Charlotte Koppe, Melissa Muszelewski, Sascha Grothe, Agnes Flöel, Marie Süße, Alexander Dressel, Felix von Podewils, Antje Vogelgesang

**Affiliations:** ^1^Department of Neurology, University Medicine, Greifswald, Germany; ^2^Department of Diagnostic Radiology and Neuroradiology, University Medicine, Greifswald, Germany; ^3^Department of Neurology, Carl-Thiem-Klinikum, Cottbus, Germany

**Keywords:** stroke, seizure, immune alterations, HMGB1, monocyte subpopulation, granulocyte subpopulation

## Abstract

**Background:** Granulocytes and monocytes are the first cells to invade the brain post stroke and are also being discussed as important cells in early neuroinflammation after seizures. We aimed at understanding disease specific and common pathways of brain-immune-endocrine-interactions and compared immune alterations induced by stroke and seizures. Therefore, we compared granulocytic and monocytic subtypes between diseases and investigated inflammatory mediators. We additionally investigated if seizure type determines immunologic alterations.

**Material and Methods:** We included 31 patients with acute seizures, 17 with acute stroke and two control cohorts. Immune cells were characterized by flow cytometry from blood samples obtained on admission to the hospital and the following morning. (i) Monocytes subpopulations were defined as classical (CD14^++^CD16^−^), (ii) intermediate (CD14^++^CD16^+^), and (iii) non-classical monocytes (CD14^dim^CD16^+^), while granulocyte subsets were characterized as (i) “classical granulocytes” (CD16^++^CD62L^+^), (ii) pro-inflammatory (CD16^dim^CD62L^+^), and (iii) anti-inflammatory granulocytes (CD16^++^CD62L^−^). Stroke patient's blood was additionally drawn on days 3 and 5. Cerebrospinal fluid mitochondrial DNA was quantified by real-time PCR. Plasma High-Mobility-Group-Protein-B1, metanephrine, and normetanephrine were measured by ELISA.

**Results:** HLA-DR expression on monocytes and their subpopulations (classical, intermediate, and non-classical monocytes) was reduced after stroke or seizures. Expression of CD32 was increased on monocytes and subtypes in epilepsy patients, partly similar to stroke. CD32 and CD11b regulation on granulocytes and subpopulations (classical, anti-inflammatory, pro-inflammatory granulocytes) was more pronounced after stroke compared to seizures. On admission, normetanephrine was upregulated in seizures, arguing for the sympathetic nervous system as inducer of immune alterations similar to stroke. Compared to partial seizures, immunologic changes were more pronounced in generalized tonic-clonic seizures.

**Conclusion:** Seizures lead to immune alterations within the immediate postictal period similar but not identical to stroke. The type of seizures determines the extent of immune alterations.

## Introduction

Tridirectional communication linking the nervous, endocrine, and immune system has become an extensively investigated scientific area in recent years (1–3). If this emerging pathophysiological concept is correct, immunological alterations may not be unique to one neurological disease but may extend to other diseases that also signal through pathways of this tridirectional communication. However, cross-study comparisons are limited, as time points of sample acquisition, assays used to determine the activation status, and determinants differ between studies. Therefore, we here compare, with an identical approach regarding sample acquisition, assays, and cytokine profiles, the cellular and humoral immunological changes induced by two neurological conditions of different etiology, i.e., seizures and stroke.

Ischemic stroke promotes so-called excitotoxic neuronal death, leading to dramatic and often irreversible loss of function. Neuroinflammation lead by granulocytes and monocytes occurs within hours after stroke onset (4–7). Following seizures, cell death is much less dramatic but is also believed to be initiated by excitotoxicity (8). Neuroinflammation is an important hallmark of epileptogenesis (9): Monocytes and granulocytes infiltrating the brain can be detected after seizures (10), and macrophages remain present in the hippocampus until chronic seizures develop in an experimental model (9). These central immunological consequences of seizures resemble findings of neuroinflammation subsequent to stroke.

Post-stroke immune alterations are thought to be induced by activation of the hypothalamic-pituitary axis (HPA) and its release of stress hormones (1, 11). Several studies have reported increased levels of epinephrine and norepinephrine or its stable metabolites metanephrine and normetanephrine after stroke (1). Increased metanephrine and normetanephrine levels during the first days after stroke are associated with lymphopenia, subsequent infections, and increased 3-month mortality rate (12, 13).

In addition, the pro-inflammatory danger-associated molecular pattern (DAMP) High-Mobility-Group-Protein B1 (HMGB-1) is thought to play an important role in the modulation of post-stroke immune alterations (12, 14). HMGB-1 binds DNA within the nucleus but can be passively released during (brain) cell death or actively secreted by immune cells as an alarmin (15). Post stroke the amount of HMGB-1 correlates rather with the amount of leukocytes in the peripheral blood than with the brain lesion size (9). Also, in epilepsy HMGB-1 is extensively discussed as an important mediator of neuroinflammation and as part of the pathophysiological mechanisms. However, our knowledge here is mainly based on animal models (16).

DAMPs are important regulators of the immune system after tissue injury and also include cell-free mitochondrial DNA (mtDNA). Release of mtDNA from the mitochondrium into the cytoplasm or into the extracellular milieu activates triggers various inflammatory pathways like inflammasome formation and cytokine production (17). Recently, mtDNA was shown to contribute to systemic inflammatory response syndrome in trauma (18) and to be increased in cerebrospinal fluid (CSF) of children with traumatic brain injury (19). Its role in stroke and seizures has not been investigated.

In stroke patients profound peripheral immune alterations associated with stroke-induced infections can be detected. Main changes include lymphocytopenia, and reduced expression of Human Leukocyte Antigen—DR isotype (HLA-DR) on monocytes (20). HLA-DR is an MHC class II cell surface receptor which is responsible for the presentation of antigens to the immune system. Presentation leads to an induction or inhibition of T-cell responses depending on the provided costimulatory signals (21).

In seizures our knowledge about functional changes of immune cell subsets in the peripheral blood is scarce. Also, only very little is known about the impact of different seizure types. The level of white blood cells is elevated in patients immediately after complex partial (PS) and/or generalized tonic-clonic seizures (GTCS) (22). Sarkis et al. found higher leukocyte counts after GTCS compared to PS, predominantly due to the increased number of monocytes (10), which is comparable to changes shortly after stroke onset (20).

After stroke and after seizures, monocytes and granulocytes are the first cell types to invade the brain from the periphery after the event. Therefore, our study compares alterations of monocytes' and granulocytes' subpopulations within the peripheral blood in both groups of patients.

CD14 gets expressed by monocytes and macrophages and helps to detect pathogens by binding lipopolysaccharides (LPS) (23). CD16 is the FcγRIII-receptor and binds IgG loaded antigens and thus initiates antibody-dependent cell-mediated cytotoxicity.

Monocytes can be divided into three different cell subpopulations by their expression of CD14 and CD16: (i) classical (CD14^++^CD16^−^), (ii) intermediate (CD14^++^CD16^+^), and (iii) non-classical monocytes (CD14^dim^CD16^+^). Classical monocytes, as the main monocyte compartment (85% of circulating monocytes), were reported to differentiate into tissue macrophages and secrete different pro- and anti-inflammatory cytokines. Intermediate (~5%) and non-classical monocytes (~10%) reveal different levels of phagocytosis and cytokine secretion, and are differentially expanded in certain diseases (24–26): While intermediate monocytes are described to produce IL-10 after LPS stimulation (27), non-classical monocytes are suggested to release tumor necrosis factor—α (TNF-α) in response to certain stimuli (23, 28). Although the percentage of classical monocytes did not change after stroke, intermediate monocytes increased and non-classical monocytes were shown to be downregulated (29, 30).

Granulocytes have similarly been subdivided into three different subpopulations (31, 32): (i) The major granulocyte subset within the peripheral blood, referred to as “classical granulocytes” (CD16^++^CD62L^+^), expresses high levels of CD16 and to some degree CD62L—a cell adhesion molecule found on leukocytes (L-selectin), (ii) pro-inflammatory granulocytes are defined as CD16^dim^CD62L^+^ cells, and (iii) anti-inflammatory granulocytes as CD16^++^CD62L^−^. Like classical monocytes, classical granulocytes develop into tissue homing granulocytes. Anti-inflammatory granulocytes display immune suppressive function after LPS stimulation and pro-inflammatory granulocytes are characterized by a high phagocytotic activity (31, 32).

In addition, the surface activation markers HLA-DR, CD32, CD62L, and CD11b were quantified on the three different subsets either monocytes or granulocytes. CD32 is a Fc receptor which mediates multiple cell-type specific functions including the release of inflammatory mediators (33) and phagocytosis (34). CD11b plays a critical role in pathogen recognition and mediates adaptive immune responses, as well as cell adhesion, endocytosis and leukocyte migration (35). To our knowledge no data exist about the regulation of these monocyte and granulocyte subsets in stroke and after seizures.

The aim of this prospective study was to investigate the following immune-parameters in a cohort of patients with a seizure disorder immediately after the epileptic seizure compared to age-matched controls: the endocrine transmitters normetanephrine, metanephrine, and the humoral pro-inflammatory DAMPs HMGB-1 and mtDNA; changes of the adaptive immune system; classical, intermediate, and non-classical subsets of monocytes as well as pro-inflammatory, anti-inflammatory, and classical subsets of granulocytes. To understand common and disease specific pathways of brain-immune-endocrine-interactions, we additionally compared the observed immune alterations induced by seizures to immune alterations found in patients after stroke. Stroke data were analyzed in regard to a second control cohort with age-matched healthy controls.

Furthermoree we investigated if the extent of immunologic alterations is determined by the occurring seizure type [complex partial (PS) and/or generalized tonic-clonic seizures (GTCS)].

## Methods

### Patients

Only patients with (1) no clinical and laboratory signs of infection (fever, symptoms of pneumonia, urinary tract infections, and other infections signs), (2) C-reactive protein (CRP) levels <50 mg/l, and (3) no immune suppressive drugs in the current medication were included. See the participants' characteristics in Table 1.

**Table 1:** Patient characteristic for adaptive and innate immune alterations.

**Patients**	**Seizure patients**				**Stroke Patients**	
**Variable**	**Total**	**Generalized tonic- clonic seizure**	**Partial seizure**	**Control**	**Total**	**Control**
*N*	31	21	10	18	17	17
Age (Years; Mean ±Std.)	56 (±19.3)	55 (±18)	60 (±21.3)	45 (±18.4)	70 (±12.1)	70 (±7.2)
Female (%)	10 (32.3%)	7 (22.6%)	3 (9.6%)	10 (55.5%)	6 (35.3%)	9 (52.9%)
Male (%)	21 (67.7%)	14 (45.2%)	7 (22.6%)	8 (44.4%)	11 (64.7%)	8 (47.1%)
Hypertension [*n* (%)]	16 (51.6%)	10 (32.3%)	6 (19.4)	4 (22.2%)	16 (94.1%)	12 (70.6%)
Dyslipidemia [*n* (%)]	8 (25.8%)	5 (16.1%)	3 (9.6%)	4 (22.2%)	9 (52.9%)	9 (52.9%)
Diabetes mellitus [*n* (%)]	4 (12.9%)	2 (6.5%)	2 (6.5%)	4 (22.2%)	3 (17.6%)	6 (35.3%)
Preexisting epilepsia [*n* (%)]	13 (42%)	6 (19,4)	7 (22.6%)	–	–	–
Hippocampal sclerosis [*n* (%)]	5 (16.1%)	4 (12,9%)	1 (3.2%)	–	–	–
NIHSS [Median (IQR)]	–	–	–	–	12 (6)	–
Treatment [*n* (%)]	–	–	–	–	15 (88.2%)	–
Systemic thrombolysis [*n* (%)]	–	–	–	–	13 (76.5%)	–
Mechanical thrombolysis [*n* (%)]	–	–	–	–	7 (41.2%)	–
Combined treatment [*n* (%)]	–	–	–	–	5 (29.4%)	–
Stroke size ccm (Mean ± Std)	–	–	–	–	83.3 (± 59.3)	–
CRP i.Pl. (mg/l) d0	7.4 (± 8.2)	6.4 (± 6.9)	9.2 (± 10.3)	5.1 (± 3.5)	11 (± 8.1)	6.8 (± 3.0)
Leukocytes (Gpt/l) d0	9.6 (± 4.5)	9.8 (± 5.1)	8.8 (± 2.5)	9.8 (± 4.2)	8.6 (± 2.1)	6.7 (± 1.3)
Thrombocytes (Gpt/l) d0	233 (± 79.7)	234.4 (± 67.8)	223.9 (± 102.1)	261.2 (± 46.6)	214.1 (± 54.1)	225.6 (± 49.0)

#### Seizure Cohort

Patients with an observed first seizure or a history of definite seizures were differentiated regarding their semiology in simple (PS) and/or complex (GTCS) seizures (included patients with generalized tonic-clonic seizures, myoclonic seizures, clonic seizures, tonic seizures, atonic seizures, typical, and atypical absences). Blood samples were taken within 24 h (h) after seizure onset (d0) and on the day thereafter (d1) (patients with CSF samples *n* = 11). Antiepileptic drugs were administered by attending physician as indicated (lamotrigine *n* = 2, valproate *n* = 3, levetiracetam *n* = 5, oxcarbamazepine *n* = 1, eslicarbazepine *n* = 1, gapapentin *n* = 1, brivaracetam *n* = 1).

#### Stroke Cohort

Blood samples were drawn within 24 h after stroke onset (d0) as well as on day 1, 3, and 5 thereafter. Patients admitted to the hospital due to ischemic middle cerebral artery occlusion within 24 h after symptom onset were eligible for the study if the National Institutes of Health Stroke Scale (NIHSS) was scored ≥ 6. Recanalization with recombinant tissue plasminogen activator (rtPA) and/or thrombectomy was carried out as clinically indicated (patients with CSF samples *n* = 8).

All patients received best medical care according to the current national guidelines and local standards.

### Controls

Two separate control cohorts were recruited for seizure and stroke patients, given different age ranges of the disease groups: Headache patients who also received CSF analysis (*n* = 13) and 3 age-matched healthy individuals from the department of ophthalmology served as controls for seizure patients (CSF samples of seizure patients: *n* = 11). Age-matched neurologically and immunologically healthy individuals from the department of ophthalmology served as controls for stroke patients. For CSF analysis, patients with tension headache (*n* = 9), unspecific dizziness (*n* = 1), premenstrual symptoms (*n* = 1), idiopathic facial paresis (*n* = 1), mild migraine (*n* = 1) served as control. Due to ethical guidelines a lumbar puncture to gain CSF cannot be carried out for research purposes only. See the participants' characteristics in Table 1 and an additional characteristics for CSF analyses in Supplementary Table 1.

### Ethics Approval and Consent to Participate

All seizure patients and controls provided direct written informed consent. Stroke patients gave written consent directly or through a surrogate. All patients and control individuals were aged ≥18 years. The study protocol was approved by the ethics committee of the Medical Faculty, University of Greifswald (No. BB 036/17 and No. BB 050/15).

### Determination of Stroke Lesion Size

Magnetic Resonance Imaging (MRI) images (3.0 Tesla) were used to calculate infarct sizes by OSIRIX 5.6. The regions of interest were defined manually, and the lesion volume was calculated semi-automatically.

### Leukocyte Subpopulations

Differential blood cell counts (XN9000, Sysmex, Norderstedt, Germany), PCT and CRP (Adivia Centaur XPT and Dimension Vista, Siemens Healthcare Diagnostics, Eschborn, Germany) were determined. Fluorescence-activated cell sorting was used to measure cell counts for leukocytes, T-cells, CD4+T-cells, CD8+T cells, NK cells and B cells as well as HLA-DR on T-cells (anti-CD45 FITC; anti-CD56 PE, anti-CD19 ECD, anti-CD3 PC5, IgG1 PC7, anti-CD4 PE, anti-CD8 PCD, anti-CD3 PC5, anti-HLA-DR-PE [FC500; Beckman Coulter]).

### Monocyte and Granulocyte Subtypes

EDTA blood was sampled and processed within 2 h to examine monocytes and granulocytes. After a red blood cell lysis using ACK lysing buffer (155 mM NH_4_Cl, 10 mM KHCO_3_, 0.1 mM EDTA), cells were stained by Zombie NIR^TM^ Fixable Viability Kit (BioLegend^TM^) on ice for 15 min to distinguish dead and alive cells, followed by a second staining with the different cell surface antibodies for 10 min on ice. Subpopulations were analyzed by flow cytometry (LSRII, BD Bioscience) [anti-HLA-DR Alexa Flour 488, anti-CD11b Brilliant Violett 421, anti-CD14 PerCP/Cy5.5, anti-CD16 Brilliant Violett 650, anti-CD62Ligand PE-Cy7, anti-CD32-PE (Biolegend)]. Death cells were distinguished by Zombie NIR TM Fixable Viability Kit (Biolegend). 200.000 events were gathered per single cell gate.

The results were evaluated using FlowJo Software 7.6.5 (Tree Star Inc.). The percentage of cells expressing a specific activation marker was determined as well as the amount of the specific marker on cell surface as defined by the mean fluorescence intensity (MFI). For the differentiation of monocytes and granulocyte subpopulation as well as activation marker fluorescence minus one controls (FMO) were used. CD14^dim^ monocytes and CD16^dim^ neutrophil population was distinguished by gating the 25th percentile of main monocyte and neutrophil population, respectively (see Supplementary Figures 1, 2 for gating strategy).

### Determination of HMGB-1

HMGB-1 was determined by ELISA according to the manufacturer's instructions (IBL, Hamburg, Germany) from plasma samples gained and immediately frozen at−80°C. Haemolytic plasma samples were excluded since they lead to false positive results. Some HMGB-1 levels exceeded the standard range and were set to the highest standard due to lack of sample for reanalysis.

### CSF Puncture and Storage

CSF sampling was done in the first 96 h of disease onset. CSF samples are obtained by lumbar puncture at the L3/L4 or L4/L5 interspaces and collected in RNAase free tubes (Nalgene, cat.no.5000–1020 and 5000–0050). Samples for routine diagnosis were frozen immediately. Material for the further analysis is immediately frozen and stored at −80°C until analysis.

### Quantification of mtDNA in CSF Samples

Total DNA was extracted from 200 μl CSF samples (Qiagen Qiamp Mini DNA Kit). After elution of DNA in 100 μl of elution buffer, the samples were concentrated by an Eppendorf concentrator to 10 μl. These samples were used in Real-Time PCR procedure for mitochondrial DNA quantification (Detroit R&D, Inc. Human DNA Damage Analysis Kit).

### Plasma Hormones

We determined normetanephrine and metanephrine, the inactive methylation products of norepinephrine and epinephrine with longer plasma half-life (36), to assess the magnitude of the adrenergic response after storage of samples at −80°C. A competitive ELISA was used according to the manufacturer instructions (MetCombi plasma ELISA; IBL, Hamburg, Germany).

### Statistical Analyses

All datasets were tested for adherence to Gaussian distribution via the Kolmogorov-Smirnov test. Multiple comparisons of Gaussian-distributed data were performed using analysis of variance; *post-hoc* analysis was performed using Bonferroni correction for multiple comparisons. Non-parametric data were analyzed by Kruskal-Wallis test with Dunn's multiple comparison test as a *post-hoc* test. *Post-hoc* tests were only performed when initial testing revealed significant differences between groups. GraphPad-PRISM 5.0 (GraphPad Software Inc., San Diego, CA, USA) was used for all analyses. A *p* < 0.05 was regarded as significant.

## Results

### Metanephrine, Normetanephrine, HMGB-1, and mtDNA in Seizure and Stroke Patients vs. Controls

Compared to controls all seizure patients showed a significantly enhanced plasma normetanephrine level on day 0 after the seizure, however, no relevant differences were found for plasma metanephrine, HMGB-1, and mtDNA in CSF (Supplementary Table 3).

Increased levels of catecholamines and HMGB-1 post-stroke are a well-documented and were also reported by our group previously (12). Of note we applied the same methods in both analyses. No alteration was observed for mtDNA in CSF of stroke patients vs. controls (Supplementary Table 3).

### Adaptive Immune Alterations in Seizure and Stroke Patients vs. Controls

**Figure 1:**
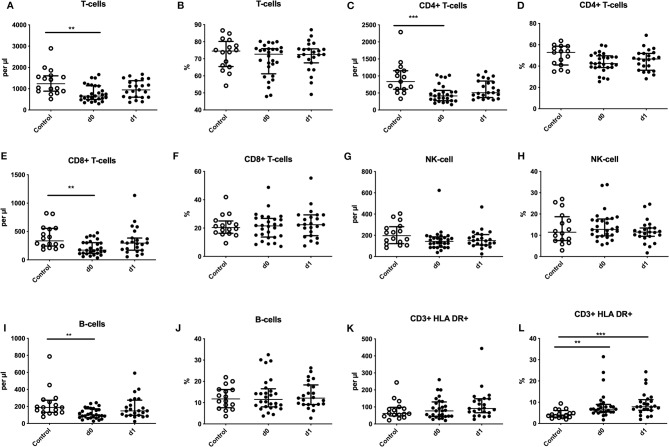
Regulation of adaptive immune cells after seizures. Absolute numbers (cells per μl) **(A,C,E,G,I,K)** and percentages **(B,D,F,H,J,L)** of T-cells **(A,B)**, CD4^+^ T-cells **(C,D)**, CD8^+^ T-cells **(E,F)**, natural killer cells (NK-cells) **(G,H)**, B-cells **(I,J)** and HLA-DR expression on T-cells **(K,L)** are shown for seizure patients on d0 and d1 (black dots) in comparison to controls (white dots). Medians and interquartile range are given. **p < 0.01; ***p < 0.001.

Absolute numbers of CD3^+^ T-lymphocytes and B-cells were significantly diminished within 24 h after the seizure (Figures 1A,I) with fast recovery on day 1. Also, absolute T-helper cells (CD4+) and cytotoxic T-cells (CD8+) were reduced on day 0 (Figures 1C,E). Percentage of HLA-DR was significantly upregulated on surface of T-lymphocytes on day 0 and day 1 (Figure 1L). These alterations of the adaptive immune system are comparable to alterations seen in stroke patients and were reported in previous publications (compare Supplementary Table 3). Percentage of T-cells, CD8+ T-cells, CD4+ T-cells, B cells and NK-cells were neither altered nor were alterations of absolute count of NK-cells and HLA-DR+ T-lymphocytes detected (Figures 1B,D,F–H,J,K).

### Monocytic Immune Alterations in Seizure and Stroke Patients vs. Controls

**Figure 2:**
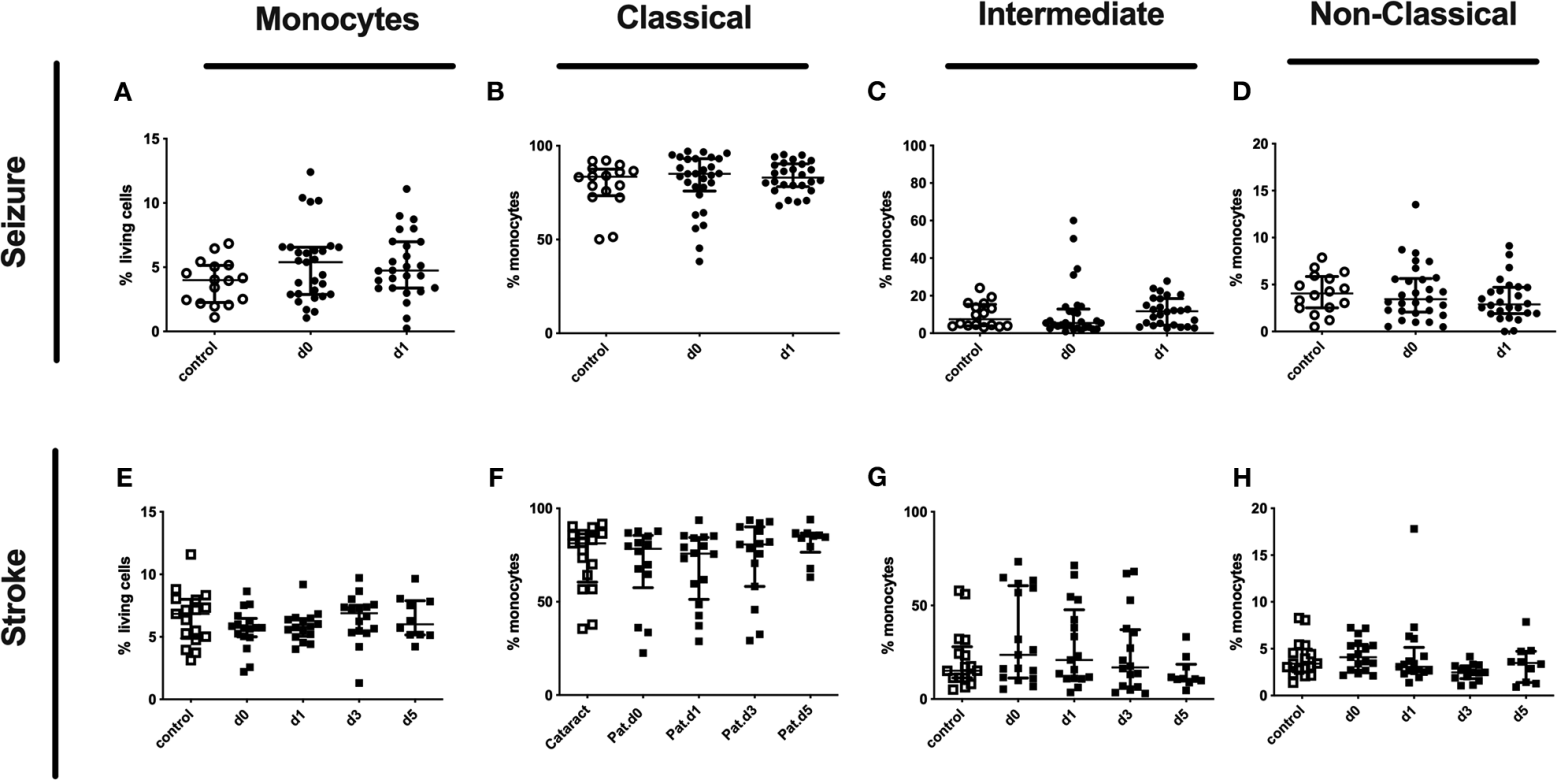
Monocytes and subpopulation in seizure and stroke patients in comparison to controls. Percentage (%) of monocytes (% living cells) **(A,E)** and classical (CD14^++^CD16^−^) **(B,F)**, intermediate (CD14^++^CD16^+^) **(C,G)** and non-classical monocytes (CD14^dim^CD16^+^) **(D,H)** is shown in patients with seizure **(A–D)** and stroke patients **(E–H)** (black dots/squares) in comparison to control patients (white dots/squares). Seizure patients were analyzed on day 0 and 1 after onset, while stroke patients were tested on day 0, 1, 3, and 5. Medians and interquartile range are given.

Percentages of classical, intermediate, and non-classical monocytes subpopulations of seizure (Figures 2A–D) and stroke patients (Figures 2E–H) were not altered in comparison to controls during our observation period.

**Figure 3:**
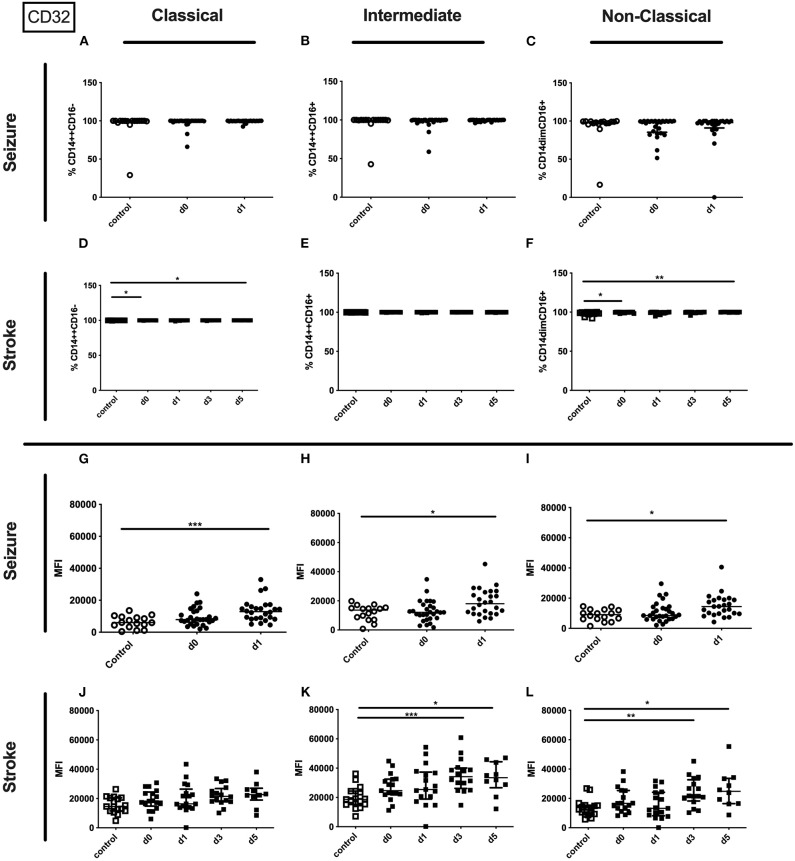
CD32 on monocyte subpopulations in seizure and stroke patients in comparison to controls. Percentage (%) **(A–F)** and expression of CD32 measured by mean fluorescence intensity (MFI) **(G–L)** was analyzed for patients with seizures **(A–C; G–I)** (black dots/squares) on day 0 and day 1 and stroke patients **(D–F,J–L)** in comparison control patients (white dots/squares). Three monocyte subpopulations were defined by CD14 and CD 16 expression—classical (CD14^++^CD16^−^) **(A,D,G,J)**, intermediate (CD14^++^CD16^+^) **(B,E,H,K)** and non-classical monocytes (CD14^dim^CD16^+^) **(C,F,I,L)**. Medians and interquartile range are given. *p < 0.05; **p < 0.01; ***p < 0.001.

While the percentage of the CD32 expressing entity of monocyte or it subpopulations was not altered (Supplementary Table 3 and Figures 3A–C), the amount of surface CD32 per cell as defined by MFI was upregulated on the entity of monocytes on day 1 after the seizure and on all subsets investigated (Figures 3G–I). In stroke patients the entity of monocytes showed increased percentage and MFI of CD32 (Supplementary Table 3). In addition the percentage of CD32 expressing classical and non-classical monocytes increased, while the amount of CD32 per cell was increased on intermediate and non-classical monocyte subsets (Figures 3D–F,J–L).

**Figure 4:**
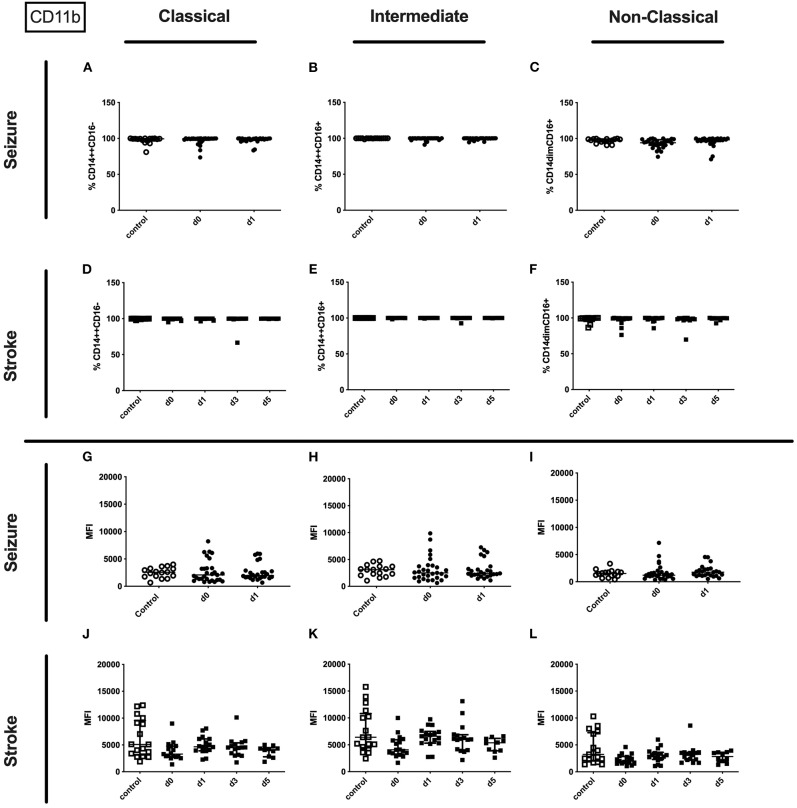
CD11b on monocyte subpopulations in seizure and stroke patients in comparison to controls. Percentage (%) **(A–F)** and expression of CD11b measured by mean fluorescence intensity (MFI) **(G–L)** was analyzed for patients with seizures **(A–C,G–I)** (black dots/squares) on day 0 and day 1 and stroke patients **(D–F,J–L)** in comparison control patients (white dots/squares). Three monocyte subpopulations were defined by CD14 and CD 16 expression—classical (CD14^++^CD16^−^) **(A,D,G,J)**, intermediate (CD14^++^CD16^+^) **(B,E,H,K)** and non-classical monocytes (CD14^dim^CD16^+^) **(C,F,I,L)**. Medians and interquartile range are given.

**Figure 5:**
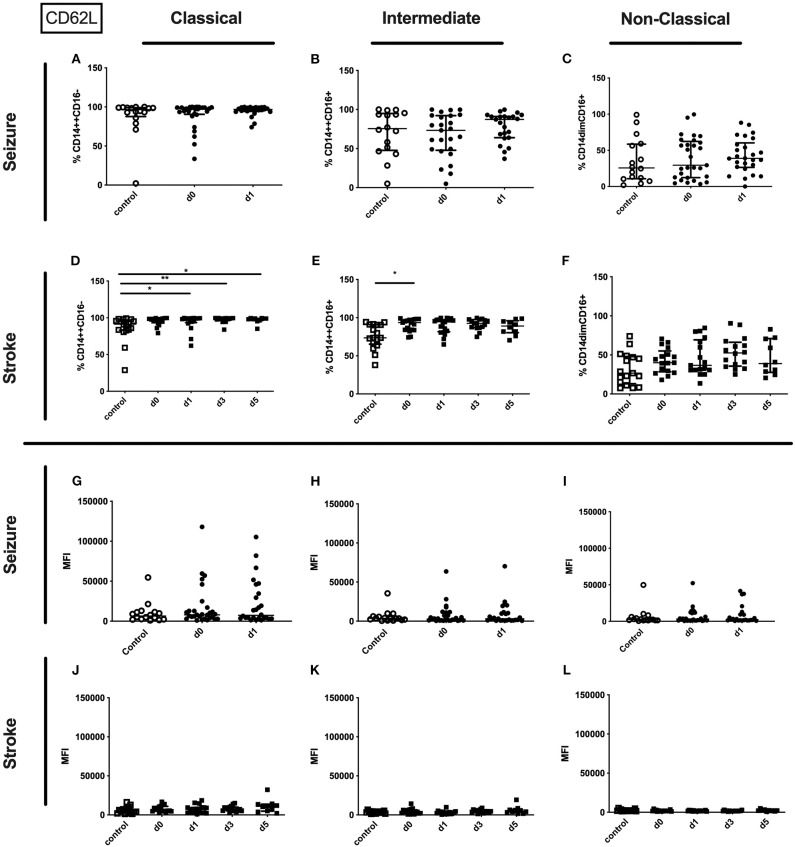
CD62L on monocyte subpopulations in seizure and stroke patients in comparison to controls. Percentage (%) **(A–F)** and expression of CD62L measured by mean fluorescence intensity (MFI) **(G–L)** was analyzed for patients with seizures **(A–C; G–I)** (black dots/squares) on day 0 and day 1 and stroke patients **(D–F,J–L)** in comparison control patients (white dots/squares). Three monocyte subpopulations were defined by CD14 and CD 16 expression—classical (CD14^++^CD16^−^) **(A,D,G,J)**, intermediate (CD14^++^CD16^+^) **(B,E,H,K)** and non-classical monocytes (CD14^dim^CD16^+^) **(C,F,I,L)**. Medians and interquartile range are given. *p < 0.05; **p < 0.01.

No alterations could be observed for CD11b (percentage and amount) for stroke and seizure patients (Figure 4). While the percentage of CD62L expressing classical and intermediate monocytes was enhanced in stroke patients only (Figures 5D–F), CD62L percentage and MFI remained unaltered in seizure patients (Figures 5A–C). MFI of CD62L was not altered in seizure and stroke patients (Figures 5G–L).

**Figure 6:**
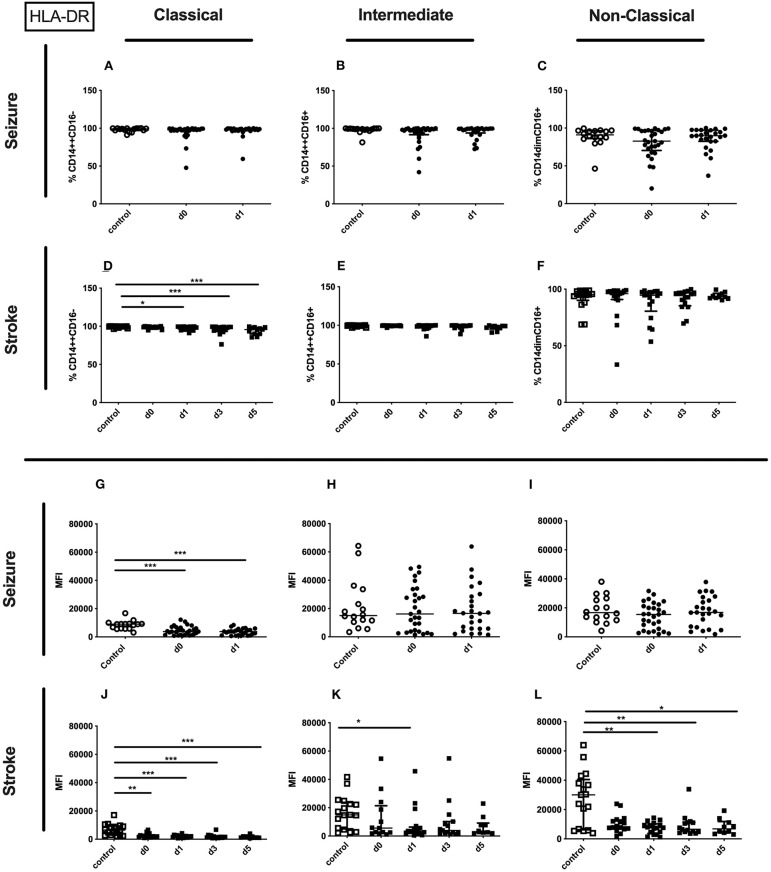
HLA-DR on monocyte subpopulations in seizure and stroke patients in comparison to controls. Percentage (%) **(A–F)** and expression of HLA-DR measured by mean fluorescence intensity (MFI) **(G–L)** was analyzed for patients with seizures **(A–C,G–I)** (black dots/squares) on day 0 and day 1 and stroke patients **(D–F,J–L)** in comparison control patients (white dots/squares). Three monocyte subpopulations were defined by CD14 and CD 16 expression—classical (CD14^++^CD16^−^) **(A,D,G,J)**, intermediate (CD14^++^CD16^+^) **(B,E,H,K)** and non-classical monocytes (CD14^dim^CD16^+^) **(C,F,I,L)**. Medians and interquartile range are given. *p < 0.05; **p < 0.01; ***p < 0.001.

The percentage of the HLA-DR expressing entity of monocytes and the amount of surface HLA-DR per monocyte were significantly downregulated on day 0 and day 1 after seizure. Also the surface amount of HLA-DR on the entity of monocytes of stroke patients was downregulated (Supplementary Table 3). These findings are based on a reduced amount of surface HLA-DR on classical monocytes in seizure patients (Figure 6G) and stroke patients (Figure 6J), and a lower amount of surface HLA-DR on non-classical monocytes in stroke patients (Figure 6L). Furthermore, the amount of surface HLA-DR was lower on intermediate monocytes on day 1 after stroke (Figure 6K). The percentage of HLA-DR was only reduced for classical monocytes in stroke patients (Figure 6D). No HLA-DR alterations were detected for further subsets in seizure and in stroke (Figures 6A–C,E,F,H,I).

### Granulocytic Immune Alterations in Seizure and Stroke Patients vs. Controls

**Figure 7:**
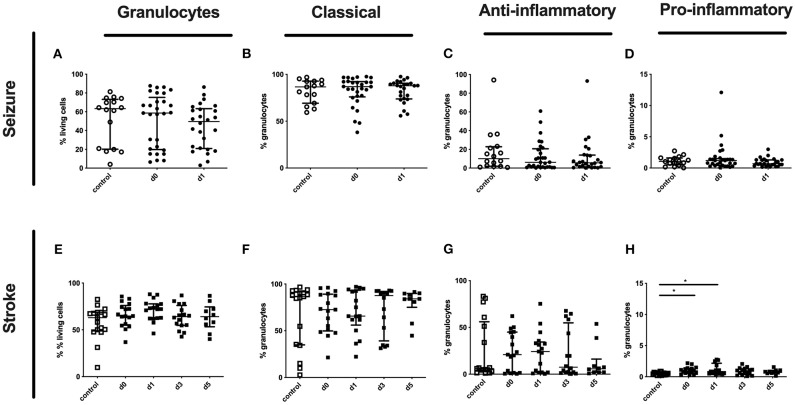
Granulocytes and subpopulation in seizure and stroke patients in comparison to controls. Percentage (%) of granulocytes (% living cells) **(A,E)** and classical (CD16^++^CD62L^+^) **(B,F)**, anti-inflammatory (CD16^++^CD62L^−^) **(C,G)** and pro-inflammatory granulocytes (CD16^dim^CD62L^+^) **(D,H)** is shown in patients with seizure **(A–D)** and stroke patients **(E–H)** (black dots/squares) in comparison to control patients (white dots/squares). Seizure patients were analyzed on day 0 and 1 after onset, while stroke patients were tested on day 0, 1, 3, and 5. Medians and interquartile range are given.

Although no altered percentages of granulocytes subpopulations were detected for seizure patients (Figures 7A–D), only pro-inflammatory granulocytes were upregulated in stroke patients (Figures 7E–H).

**Figure 8:**
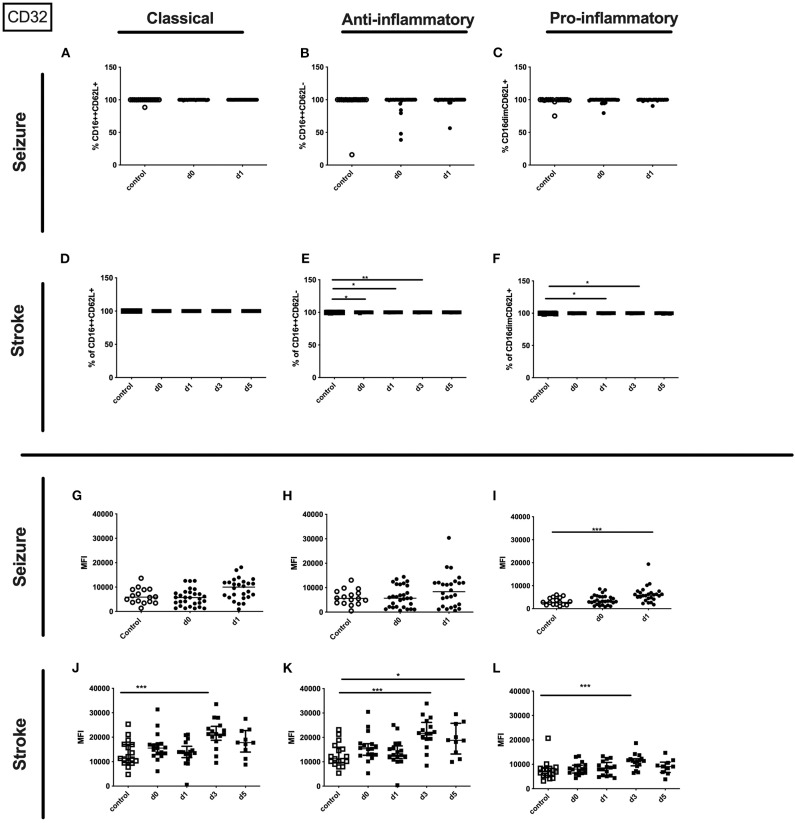
CD32 on granulocyte subpopulations in seizure and stroke patients in comparison to controls. Percentage (%) **(A–F)** and expression of CD32 measured by mean fluorescence intensity (MFI) **(G–L)** was analyzed for patients with seizures **(A–C,G–I)** (black dots/squares) on day 0 and day 1 and stroke patients **(D–F,J–L)** in comparison control patients (white dots/squares). Three granulocyte subpopulations were defined by CD16 and CD62L expression—classical (CD16^++^CD62L^+^) **(A,D,G,J)**, anti-inflammatory (CD16^++^CD62L^−^) **(B,E,H,K)** and pro-inflammatory granulocytes (CD16^dim^CD62L^+^) **(C,F,I,L)**. Medians and interquartile range are given. *p < 0.05; **p < 0.01; ***p < 0.001.

In seizure the surface amount of CD32 on the entity granulocytes was significantly increased in patients' blood on day 1 after the seizure compared to controls (Supplementary Table 3). In addition, the surface amount of CD32 was increased on the pro-inflammatory subset on day 1 in seizure patients (Figure 8I). This tendency was also observed for classical and anti-inflammatory granulocytes in the seizure group (Figures 8G,H).

In stroke patients surface amount of CD32 was enhanced on the entity of granulocytes and especially on day 5 after stroke on the three different granulocyte subpopulations (Supplementary Table 3 and Figures 8J–L).

Only the percentage of CD32 expressing pro- and anti-inflammatory granulocytes was upregulated after stroke (Figures 8D–F). Percentage of CD32 on granulocyte subsets was not altered in seizure patients (Figures 8A–C).

**Figure 9:**
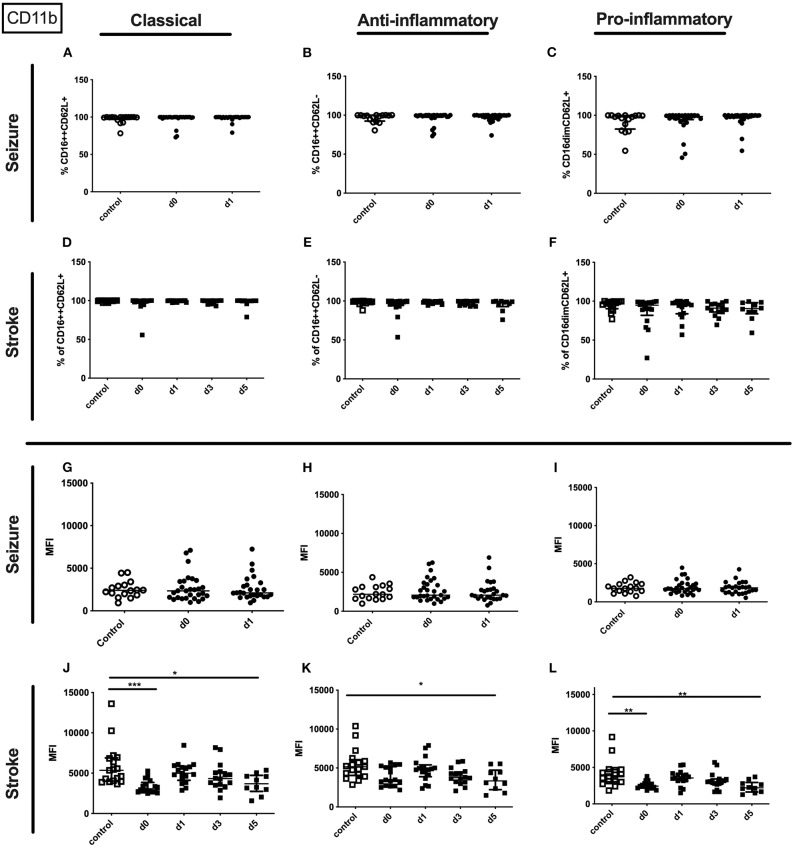
CD11b on granulocyte subpopulations in seizure and stroke patients in comparison to controls. Percentage (%) **(A–F)** and expression of CD11b measured by mean fluorescence intensity (MFI) **(G–L)** was analyzed for patients with seizures **(A–C,G–I)** (black dots/squares) on day 0 and day 1 and stroke patients **(D–F,J–L)** in comparison control patients (white dots/squares). Three granulocyte subpopulations were defined by CD16 and CD62L expression—classical (CD16^++^CD62L^+^) **(A,D,G,J)**, anti-inflammatory (CD16^++^CD62L^−^) **(B,E,H,K)** and pro-inflammatory monocytes (CD16^dim^CD62L^+^) **(C,F,I,L)**. Medians and interquartile range are given. *p < 0.05; **p < 0.01; ***p < 0.001.

The entity of granulocytes did not display altered CD11b percentage or amount in seizure patients. In contrast the amount of surface CD11b on the entity of granulocytes in stroke patients was lowered on day 0 and day 5 (Supplementary Table 3). This finding was partly resembled in the surface CD11b amount on all three granulocyte subpopulations (Figures 9J–L) in stroke patients' while not CD11b alterations were observed in seizure patients granulocyte subpopulations (Figures 9A–C,G–I). Also, percentage of CD11b was not altered for all granulocyte subpopulations in stroke patients (Figures 9D–F).

Metanephrine, normetanephrine, HMGB-1, and mtDNA of partial seizure (PS) vs. generalized tonic-clonic seizure patients (GTCS).

Levels of metanephrine, normetanephrine, or HMGB-1 and mtDNA levels were not significantly different when comparing PS and GTCS (Supplementary Table 2).

### Adaptive Immune Alterations of PS vs. GTCS

No significant differences were observed for T-lymphocytes, CD4^+^ and CD8^+^ T-cells, NK-cells, B-cells and the percentage of HLA-DR expressing CD3^+^ cells (Supplementary Table 2).

### Monocytic Immune Alterations of PS vs. GTCS

**Figure 10:**
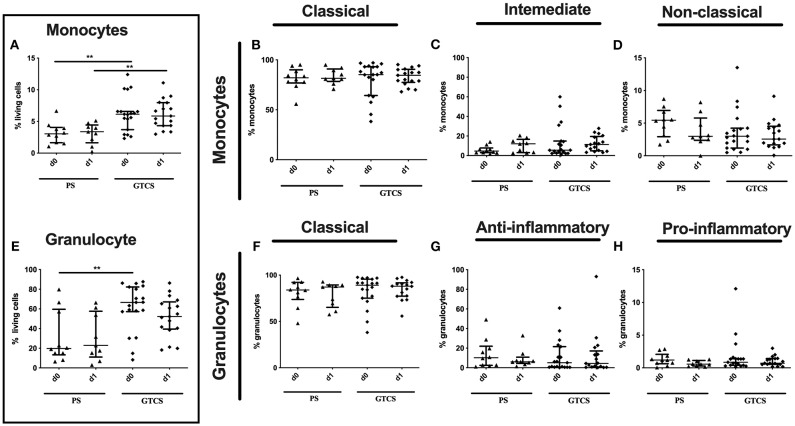
Monocytes and granulocytes subpopulations of patients with PS vs. GTCS. Percentage (%) of monocytes (% living cells) **(A)** and classical (CD14^++^CD16^−^) **(B)**, intermediate (CD14^++^CD16^+^) **(C)** and non-classical monocytes (CD14^dim^CD16^+^) **(D)** as well as granulocytes (% living cells) **(E)** and classical (CD16^++^CD62L^+^) **(F)**, anti-inflammatory (CD16^++^CD62L^−^) **(G)** and pro-inflammatory granulocytes (CD16^dim^CD62L^+^) **(H)**. Seizure patients are divided into patients with partial seizures (PS) (black triangle) and generalized tonic-clonic seizures (GTCS) (black squares) on day 0, 1 of seizure onset. Medians and interquartile range are given. **p < 0.01.

The percentage of the entity of monocytes was upregulated after GTCS in comparison to PS without any alterations of subpopulations (Figures 10A–D).

**Figure 11:**
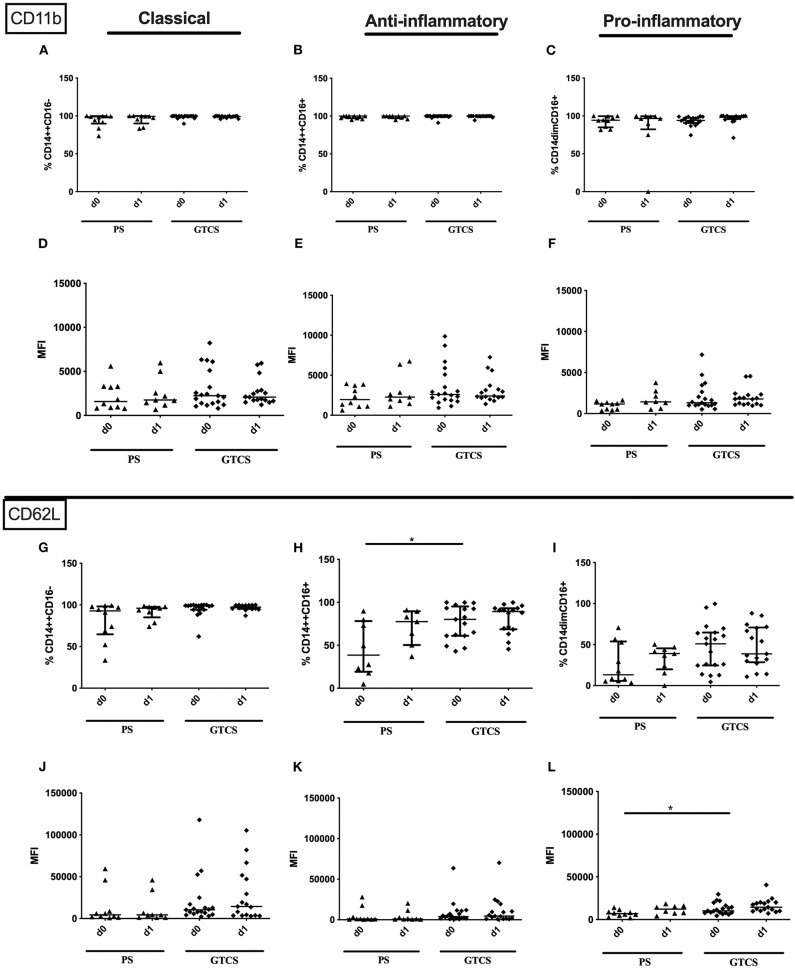
CD11b and CD62L on monocyte subpopulations of patients with PS vs. GTCS. Percentage (%) **(A–C,G–I)** and expression of CD11b **(A–F)** and CD62L **(G–L)** measured by mean fluorescence intensity (MFI) **(D–F, J–L)** was analyzed for with partial seizures (PS) (black triangle) and generalized tonic-clonic seizures (GTCS) (black squares) on day 0, 1 of seizure onset. Three monocyte subpopulations were defined by CD14 and CD 16 expression—classical (CD14^++^CD16^−^) **(A,D,G,J)**, intermediate (CD14^++^CD16^+^) **(B,E,H,K)** and non-classical monocytes (CD14^dim^CD16^+^) **(C,F,I,L)**. Medians and interquartile range are given. *p < 0.05.

**Figure 12:**
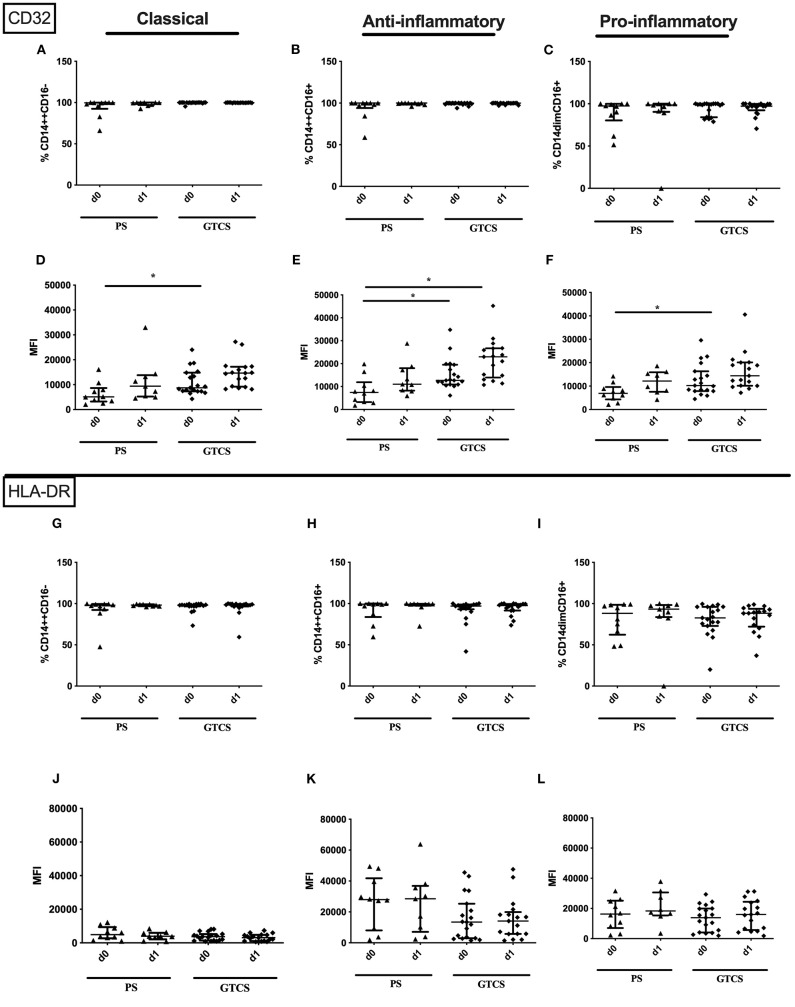
CD32 and HLA-DR on monocyte subpopulations of patients with PS vs. GTCS. Percentage (%) **(A–C,G–I)** and expression of CD32 **(A–F)** and HLA-DR **(G–L)** measured by mean fluorescence intensity (MFI) **(D–F,J–L)** was analyzed for with partial seizures (PS) (black triangle) and generalized tonic-clonic seizures (GTCS) (black squares) on day 0, 1 of seizure onset. Three monocyte subpopulations were defined by CD14 and CD 16 expression—classical (CD14^++^CD16^−^) **(A,D,G,J)**, intermediate (CD14^++^CD16^+^) **(B,E,H,K)** and non-classical monocytes (CD14^dim^CD16^+^) **(C,F,I,L)**. Medians and interquartile range are given. *p < 0.05.

Compared to PS several alterations could be found following GTCS: (i) the percentage of CD62L expressing monocytes in general (Supplementary Table 2) as well as the intermediate subpopulation (Figure 11H) was increased, (ii) the amount of surface CD62L was higher on non-classical monocytes on d1 (Figure 11L); (iii) the amount of CD32 on the entity of monocytes (Supplementary Table 2) and on all three subpopulations was increased (Figures 12D–F). CD11b (Figures 11A–F), and HLA-DR (Figures 12G–L) was not altered within PS and GTCS. No other alterations were detected for monocyte activation marker (CD32, CD11b and HLA-DR) in comparison of PS to GTCS (Figures 11G,I–L, 12A–C).

### Granulocytic Immune Alterations of PS vs. GTCS

**Figure 13:**
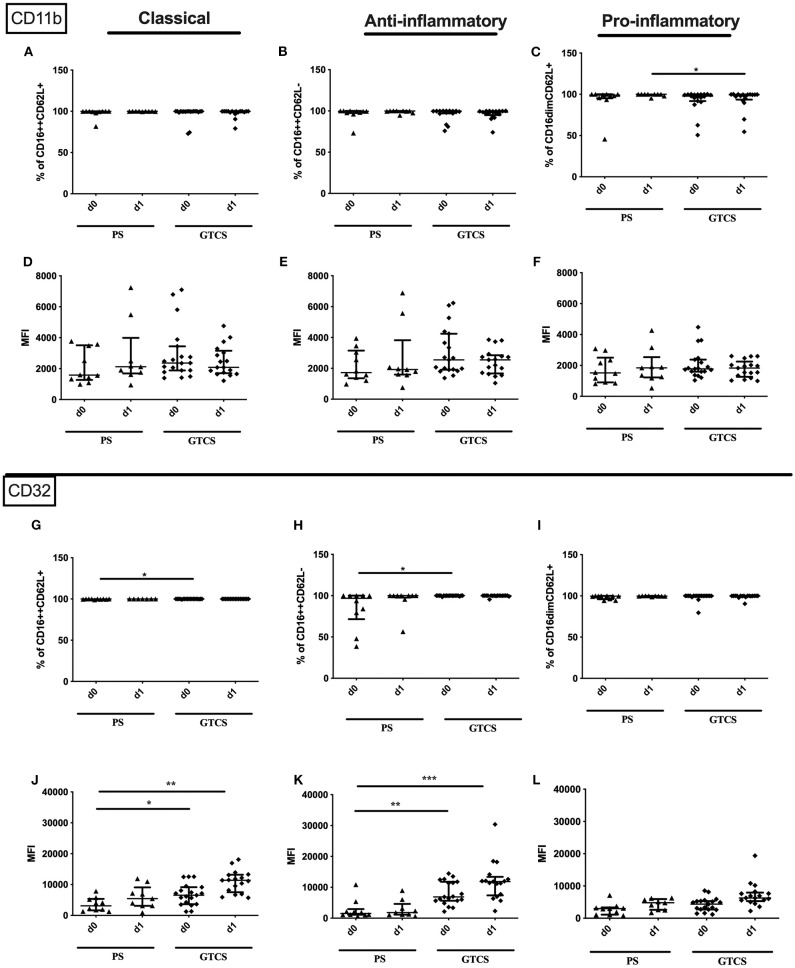
CD11b and CD32 on granulocytes subpopulations of patients with PS vs. GTCS. Percentage (%) **(A–C,G–I)** and expression of CD11b **(A–F)** and CD32 **(G–L)** measured by mean fluorescence intensity (MFI) **(D–F,J–L)** was analyzed for with partial seizures (PS) (black triangle) and generalized tonic-clonic seizures (GTCS) (black squares) on day 0, 1 of seizure onset. Three granulocyte subpopulations were defined by CD16 and CD62L expression—classical (CD16^++^CD62L^+^) **(A,D,G,J)**, anti-inflammatory (CD16^++^CD62L^−^) **(B,E,H,K)** and pro-inflammatory granulocytes (CD16^dim^CD62L^+^) **(C,F,I,L)**. Medians and interquartile range are given. Medians and interquartile range are given. *p < 0.05; **p < 0.01; ***p < 0.001.

Compared to PS the following alteration could be found after GTCS: (i) the percentage of the entity of granulocytes was higher on day 0 (Figure 10E), (ii) percentages and the amounts of CD32^+^ granulocytes (Supplementary Table 2) as well as of CD32 expressing classical and anti-inflammatory cells were higher (Figures 13G,H,J,K). The percentage of CD11b expressing pro-inflammatory granulocytes was increased (Figure 13C). No other alterations were detected for overall granulocyte subpopulations (Figures 10F–H) and their activation marker (CD32, CD11b) in comparison of PS to GTCS (Figures 13A,B,D–F,I,L).

## Discussion

Blood brain barrier disruption, subsequent neuroinflammation, and post-stroke immune alteration are well-described phenomena of ischemic stroke (4, 5). While neuroinflammation has also been studied in epilepsy, the knowledge about the impact of seizures on major immune cell populations is scarce. To our knowledge this is the first study to provide quantitative and qualitative insight into cellular adaptive and innate immune alterations after different types of seizures: PS and GTCS seizures. Moreover, we were able to detect novel similarities to post-stroke immune alterations in a side by side comparison of the two diseases.

### Epinephrine, HMGB-1 Induce Immune Alterations in Stroke, but Only Norepinephrine Was Upregulated for Seizures

Our seizure cohort showed an induction of the HPA by the release of norepinephrine. These findings are in line with Bauer et al. who reported a raise of epinephrine levels after seizure in average by 454% (37). The HPA was also identified to transduce stroke induced immune alterations (12, 14, 38). In both diseases the HPA seems to be an important mediator of subsequent immune alterations while it remains unclear which mechanisms are needed to maintain immunosuppression for several days.

In contrast to post-stroke alterations, where HMGB-1 increase is well-documented (12, 39, 40), we could not detect any alterations of HMGB-1 after seizures. Note that the same ELISA was used in both groups. HMGB-1 can be released actively by immune cells and passively by dying cells these. Depending on the pathophysiological mechanism different HMGB-1 isoforms are released (41). The ELISA used in our study is not able to detect these different isoforms. While there is evidence for an important pathophysiological role of HMGB-1 in epilepsy from several studies applying animal models as reviewed in Paudel et al. (42), there is only one paper reporting increased disulfide HMGB-1 in epilepsy patients (43). Additional experiments should clarify the role of HMGB-1 in epilepsy patients and also analyze different isoforms.

### Seizure and Stroke Induced Alterations of Activation Marker in Monocyte/Granulocyte Subsets

The increase of monocytes in our seizure cohort is in line with the work of Sarkis et al. this group found a higher number of monocytes in patients with GTCS compared to those with partial seizures (10). We found an upregulation of the amount of CD32 for granulocytes and monocytes as well as a reduced amount of HLA-DR on monocytes in stroke and seizure patients. These findings were partly resembled in their subpopulations. Stroke patients showed a pronounced upregulation of CD32 and downregulation of HLA-DR. The amount of CD11b was reduced in granulocytes and granulocyte subpopulations of stroke patients, while no alterations were observed for seizure patients. CD32 is an FcyII receptor which binds complexed IgG. The phagocytosis of immune complex-opsonized by human macrophages is substantially enhanced (44). Upregulated CD32 was shown to be essential for bacterial defense depending on its polymorphism (45). HLA-DR expression was reduced in general on monocytes and on the classical monocytes in patients suffering from seizures.

### Immune Alterations in PS and GTCS

Granulocytosis, monocytosis, and different activation markers were especially pronounced in GTCS in comparison to partial seizures. In addition, we were able to detect differences in the activation state of cells. The magnitude of immune regulation seems to be a sequel of acute epileptic activity and is influenced by seizure duration and semiology. While epileptic activity in PS has a regional or hemispheric distribution, GTCS affects both brain hemispheres (46), this might explain a major impact of GTCS seizures on brain-immune-interaction.

### Limitations

First, this is a single-center study with a relatively small group of patients with acute seizures, limiting the statistical validity of our data, particularly with regard to subgroup analyses such as seizure types. Since seizure patients were younger than stroke patients, we recruited two age-matched control groups. Therefore, differences regarding sex and comorbidities may have influenced immune alterations. However, this exploratory analysis is the first to describe the influence of seizure type on immunological alterations which should be investigated in depth in future studies. Here we investigated alterations in the peripheral blood, immune alterations in the intrathecal compartment cannot be detected by this approach. Experimental animal models will be required to investigate migration patterns and activation status of granulocytes and monocytes.

Second, although several patients were treated with antiepileptic drugs, the small sample size precludes further analyses on the impact of different drugs on immune alterations. Still, an exploratory subanalysis of antiepileptic drugs with known immunomodulatory properties [by levetiracetam, valproate, gabapentin, lamotrigine, oxcarbamazepine (47–52)] compared to untreated patients did not reveal differences. Third, our study provides solely descriptive data. In further studies the importance of the observed immune alterations for disease pathology or treatment options has to be clarified by taking advantage of animal models and intervention strategies.

## Summary and Conclusions

**Figure 14:**
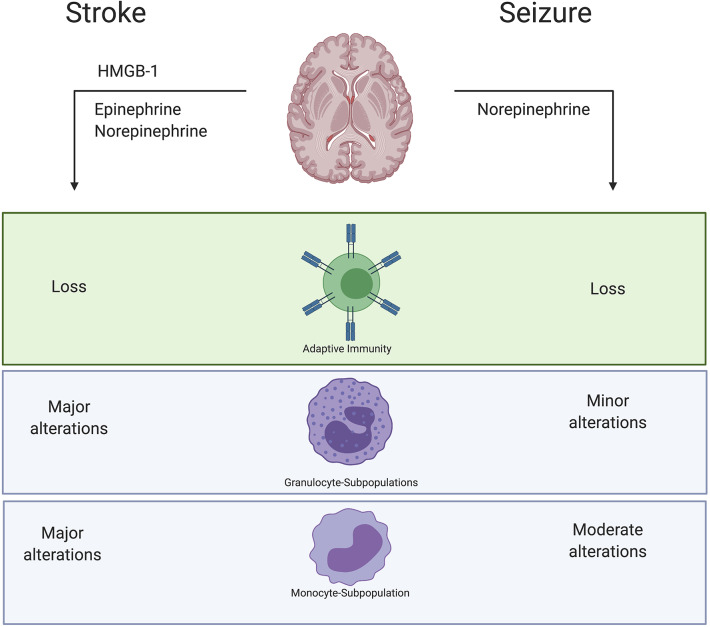
Summary results. Stroke causes a release of Epinephrine and Norepinephrine as well as death associated molecular patterns (DAMPS) like HMGB-1. Also, in seizure we could verify a release of Norepinephrine. However, immune alterations within the immediate postictal period after seizure are similar but not identical to stroke. Especially alterations of monocytes- and granulocyte-subtypes seem to be more pronounced regulated after stroke compared to acute seizures. Described immune alterations were observed in peripheral blood samples.

It seems that common pathways of brain-immune-interactions are at play in stroke as well as in seizures and might also exert similar impact on the peripheral immune response (Figure 14): a loss of lymphocytes and granulocytosis as well as a reduced HLA-DR expression on monocytes and their subpopulations. However, immune alterations within the immediate postictal period after seizure are similar but not identical to stroke. The HPA is activated and consequently triggers neuroinflammation and changes in peripheral immune response in both conditions. Especially alterations of monocyte- and granulocyte-subtypes seem to be more profoundly regulated after stroke compared to seizures. While about half of the body's monocytes are stored as a reserve in the spleen, granulocytes are recruited from bone marrow. Our results may thus suggest that acute brain damage induced by stroke induces stronger changes in bone marrow, possibly by direct nerval innervation. The temporary dysfunction with little permanent tissue damage might not be sufficient to induce these changes. Since our study cohort provides a unique insight into the differences between PS and GTCS, the semiological subtypes might determine the range of immune modulations.

A direct comparison of immune alterations in various neurological diseases is required to detect common therapeutic targets to prevent deleterious immune alterations and improve patient outcome across a wide spectrum of acute diseases.

## Data Availability Statement

The datasets acquired during and/or analyzed during the current study are available from the corresponding author upon reasonable request.

## Ethics Statement

The studies involving human participants were reviewed and approved by the ethics committee of the Medical Faculty, University of Greifswald (No. BB 036/17 and No. BB 050/15). The patients/participants provided their written informed consent to participate in this study.

## Author Contributions

AD, JR, AV, MS, and FP substantially contributed to the conception and design of the study. JT, SC, MM, and CK acquired the data and technically analyzed them. SG provided stroke lesion sizes. AV, JR, AD, and FP interpreted the data. AV and JR drafted the manuscript. AD, AF, and FP revised it critically for important intellectual content. All authors have read and approved the final manuscript.

## Conflict of Interest

The authors declare that the research was conducted in the absence of any commercial or financial relationships that could be construed as a potential conflict of interest.

## Supplementary Material

Supplementary Table 1 Patients characteristic for CSF samples.

Supplementary Table 2 Immune alterations in patients suffering from a partial seizure in comparison to patients with tonic-clonic events are shown. Significance level (^*^*p* < 0.05; ^**^*p* < 0.01; ^***^*p* < 0.001), p-value for ANOVA or Kruskal Wallis test and regulation (Upregulation ↑; Reduction ↓) are given.

Supplementary Table 3 Immune alterations of stroke and seizure cohort are shown. Significance level (^*^*p* < 0.05; ^**^*p* < 0.01; ^***^*p* < 0.001), p-value for ANOVA or Kruskal Wallis test and regulation (Upregulationt ↑; Reduction ↓) are given.

Supplementary Figure 1A Gating strategy for granulocyte subpopulations. Representative probe for activation marker on granulocyte subpopulation. After single cell gating **(A)** and determination of living cells by ZOMBIE **(B)**, cells were gated by SSC-A **(C)** and subpopulation marker CD62L in their subpopulation according to FMO **(D)**. CD16^dim^ neutrophil population **(E)** was distinguished by gating the 25th percentile of main neutrophil population.

Supplementary Figure 1B Gating strategy for monocyte subpopulations: Representative probe for activation marker on granulocyte subpopulation. After single cell gating **(A)** and determination of living cells by ZOMBIE **(B)**, cells were gated by SSC-A **(C)** and subpopulation marker (CD14) in their subpopulation according to FMO **(D)**. CD14^dim^ monocytes **(E)** population was distinguished by gating the 25th percentile of main neutrophil population.
