# Supplementary material for: Immune Alterations Following Neurological Disorders: A Comparison of Stroke and Seizures
Source: Front Neurol. 2020 Jun 2;11:425. doi: 10.3389/fneur.2020.00425 (PMC7280464; doi:10.3389/fneur.2020.00425)
Supplement: Supplementary Table 1 — Patients characteristic for CSF samples. [file Data_Sheet_1.PDF]

Supplemental Table 1

Patient Characteristic for CSF Analysis

	Seizure patients					Stroke Patients
Variable	Total	Generalized tonic- clonic seizure	partial seizure	control		Total
n (%)	11	10	1	16		8
Age (Years; Mean ±Std.)	53 (±18,3)	51,5 (17,5)	78	42(±16,0)		42 (±5,8)
female (%)	9 (81,8%)	9 (81,8%)	0	11 (68,7%)		6 (75%)
male (%)	2 (18,2%)	1 (9,1%)	1 (9,1%)	5 (31,3%)		2 (25%)
Hypertension (n (%))	5 (45,5%)	4 (45,5 %)	0	2 (12,5%)		5 (62,2%)
Dyslipidemia (n (%))	2 (18,1%)	1 (9,05%)	1 (9,05%)	2 (12,5%)		2 (25%)
Diabetes mellitus (n (%))	0	0	0	1 (6,3%)		0
CRP i.Pl. (mg/l) d0	7,5 (± 8,8)	8,0 (± 9,1)	3,1	5,1 (± 3,5)		21,7 (± 16,0)
Leukocytes (Gpt/l) d0	7,8 (± 2,8)	7,9 (± 3,0)	7	9,8 (± 4,2)		8,3 (± 1,7)
Thrombocytes (Gpt/l) d0	211 (± 57,9)	220 (± 53,1)	123	261,2 (± 46,6)		264 (± 46,6)
