# Supplementary material for: Immune Alterations Following Neurological Disorders: A Comparison of Stroke and Seizures
Source: Front Neurol. 2020 Jun 2;11:425. doi: 10.3389/fneur.2020.00425 (PMC7280464; doi:10.3389/fneur.2020.00425)
Supplement: Supplementary Table 2 — Immune alterations in patients suffering from a partial seizure in comparison to patients with tonic-clonic events are shown. Significance level (*p < 0.05; **p < 0.01; ***p < 0.001), p-value for ANOVA or Kruskal Wallis test and regulation (Upregulation ↑; Reduction ↓) are given. [file Data_Sheet_2.PDF]

partial vs. generalized tonic-clonic seizures	d0 vs. d0	d1 vs. d1	p -value	Regulation (↑↓)
Adaptive Immune cells				
Normetanephrine	-	-	-	
Metanephrin	-	-	-	
HMGB1	-	-	-	
**T-cell (%)**	-	-	-	
**T-cell (per µl)**	-	-	-	
**CD4+ T-cell (%)**	-	-	-	
**CD4+ T-cell (per µl)**	-	-	-	
**CD8+ T-cell (%)**	-	-	-	
**CD8+ T-cell (per µl)**	-	-	-	
**NK-cell (%)**	-	-	-	
**NK-cell (in µl)**	-	-	-	
**B-cell (%)**	-	-	-	
**B-cell (per µl)**	-	-	-	
HLA-DR+ CD3+ T- cell (%)	-	-	-	
HLA-DR+ CD3+ T- cell (per µl)	-	-	-	
MtDNA in CSF	-	-	-	
Innate Immune cells				
Granulocytes				
**all granulocytes (%)**	**	-	**0.0051**	↑
CD11b+ (%)	-	-	-	
CD11b+ (MFI)	-	-	-	
CD32+ (%)	**	-	0.0035	↑
CD32+ (MFI)	*	*	0.001	↑
**classical_CD16++ CD62L+ (%)**	-	-	-	
CD16++ CD62L+\ CD11b+ (%)	-	-	-	
CD16++CD62L+ \CD11b+ (MFI)	-	-	-	
CD16++CD62L+\ CD32+ (%)	*	-	0.0259	↑
CD16++CD62L+\ CD32+ (MFI)	*	**	< 0.0001	↑
**anti-inflammatory_CD16++CD62L- (%)**	-	-	-	
CD16++CD62L-\ CD11b+ (%)	-	-	-	
CD16++CD62L-\ CD11b+ (MFI)	-	-	-	
CD16++CD62L-\ CD32+ (%)	*	-	0.0543	↑
CD16++CD62L-\ CD32+ (MFI)	**	***	< 0.0001	↑
**pro-inflammatory_CD16dim CD62L+ (%)**	-	-	-	
CD16dim CD62L+\ CD11b+ (%)	-	*	0.0264	↑
CD16dim CD62L+\ CD11b+ (MFI)	-	-	-	
CD16dim CD62L+\ CD32+ (%)	-	-	-	
CD16dim CD62L+\ CD32+ (MFI)	-	-	-	
Monocytes				
**All monocytes (%)**	**	**	**0.0004**	↑
CD11b+ (%)	-	-	-	
CD11b+ (MFI)	-	-	-	
CD62L+ (%)	*	-	0.0433	↑
CD62L+ (MFI)	-	-	-	
CD32+ (%)	-	-	-	
CD32+ (MFI)	*	-	0.0114	↑
HLA-DR+ (%)	-	-	-	
HLA-DR+ (MFI)	-	-	-	
**classical_ CD14++ CD16- (%)**	-	-	-	
CD14++CD16-\CD11b+ (%)	-	-	-	
CD14++CD16-\ CD11b+ (MFI)	-	-	-	
CD14++CD16-\ CD32+ (%)	-	-	-	
CD14++CD16- \ CD32+ (MFI)	*	-	0.0018	↑
CD14++CD16-\ CD62L+ (%)	-	-	-	
CD14++CD16- \ CD62L+ (MFI)	-	-	-	
CD14++CD16-\ HLA-DR+ (%)	-	-	-	
CD14++CD16- \ HLA-DR+ (MFI)	-	-	-	
**anti-inflammatory_CD14++CD16+ (%)**	-	-	-	
CD14++CD16+\ CD11b+ (%)	-	-	-	
CD14++CD16+\CD11b+ (MFI)	-	-	-	
CD14++CD16+\ CD32+ (%)	-	-	-	
CD14++CD16+\ CD32+ (MFI)	*	*	0.0003	↑
CD14++CD16+\ CD62L+ (%)	*	-	0.0351	↑
CD14++CD16+\ CD62L+ (MFI)	-	-	-	
CD14++CD16+\ HLA-DR+ (%)	-	-	-	
CD14++CD16+\ HLA-DR+ (MFI)	-	-	-	
**pro_inflammatory_CD14dim CD16+ (%)**	-	-	-	
CD14dim CD16+\CD11b+ (%)	-	-	-	
CD14dim CD16+ \ CD11b+ (MFI)	-	-	-	
CD14dim CD16+\ CD32+ (%)	-	-	-	
CD14dim CD16+ \ CD32+ (MFI)	*	-	0.0049	↑
CD14dim CD16+\ CD62L+ (%)	-	-	-	
CD14dim CD16+ \ CD62L+ (MFI)	-	**	0.0084	↑
CD14dim CD16+\ HLA-DR+ (%)	-	-	-	
CD14dim CD16+ \ HLA-DR+ (MFI)	-	-	-	
