# Supplementary material for: Immune Alterations Following Neurological Disorders: A Comparison of Stroke and Seizures
Source: Front Neurol. 2020 Jun 2;11:425. doi: 10.3389/fneur.2020.00425 (PMC7280464; doi:10.3389/fneur.2020.00425)
Supplement: Supplementary Table 3 — Immune alterations of stroke and seizure cohort are shown. Significance level (*p < 0.05; **p < 0.01; ***p < 0.001), p-value for ANOVA or Kruskal Wallis test and regulation (Upregulationt ↑; Reduction ↓) are given. [file Data_Sheet_3.PDF]

	stroke						seizure			
	ctrl. vs. d0	ctrl. vs. d1	ctrl. vs. d3	ctrl. vs. d5	p-value	Regulation (↑↓)	ctrl. vs. d0	ctrl. vs. d1	p-value	Regulation (↑↓)
Adaptive immune cells										
Normetanephrine	-	-	-	-	-	↑[1]	*	-	0.0274	↑
Metanephrine	-	-	-	-	-	↑[1]	-	-	-	-
HMGB1	-	-	-	-	-	↑[1]	-	-	-	-
**T-cell (%)**	-	-	-	-	-	↓[1]	-	-	-	-
**T-cell (per µl)**	-	-	-	-	-	↓[1]	**	-	**0.0046**	↓
**CD4+ T-cell (%)**	-	-	-	-	-	↓[1]	-	-	-	-
**CD4+ T-cell (per µl)**	-	-	-	-	-	↓[1]	***	-	**0.001**	↓
**CD8+ T-cell (%)**	-	-	-	-	-	↓[1]	-	-	-	-
**CD8+ T-cell (per µl)**	-	-	-	-	-	↓[1]	**	-	**0.0046**	↓
**NK-cell (%)**	-	-	-	-	-	-	-	-	-	-
**NK-cell (per µl)**	-	-	-	-	-	-	-	-	-	-
**B-cell (%)**	-	-	-	-	-	-	-	-	-	-
**B-cell (per µl)**	-	-	-	-	-	-	**	-	**0.0034**	↓
HLA-DR+ CD3+ T- cell (%)	-	-	-	-	-	-	**	***	0.0007	↑
HLA-DR+ CD3+ T- cell (per µl)	-	-	-	-	-	-	-	-	-	-
MtDNA in CSF	-	-	-	-	-	-	-	-	-	-
Innate immune cells										
Granulocytes										
**all granulocytes (%)**	-	-	-	-	-	-	-	-	-	-
CD11b+ (%)	-	-	-	-	-	-	-	-	-	-
CD11b+ (MFI)	***	-	-	*	0.0002	↓	-	-	-	-
CD32+ (%)	-	-	-	-	-	-	-	-	-	-
CD32+ (MFI)	-	-	***	-	0.0005	↑	-	*	0.014	↑
**classical_CD16++ CD62L+ (%)**	-	-	-	-	-	-	-	-	-	-
CD16++ CD62L+\ CD11b+ (%)	-	-	-	-	-	-	-	-	-	-
CD16++CD62L+ \CD11b+ (MFI)	***	-	-	*	< 0.0001	↓	-	-	-	-
CD16++CD62L+\ CD32+ (%)	-	-	-	-	-	-	-	-	-	-
CD16++CD62L+\ CD32+ (MFI)	-	-	***	-	0.0006	↑	-	-	-	-
**anti-inflammatory_CD16++CD62L- (%)**	-	-	-	-	-	-	-	-	-	-
CD16++CD62L-\ CD11b+ (%)	-	-	-	-	-	-	-	-	-	-
CD16++CD62L-\ CD11b+ (MFI)	-	-	-	*	0.0181	↓	-	-	-	-
CD16++CD62L-\ CD32+ (%)	*	*	**	-	0.0119	↑	-	-	-	-
CD16++CD62L-\ CD32+ (MFI)	-	-	***	*	0.0004	↑	-	-	-	-
**pro-inflammatory_CD16dim CD62L+ (%**	*	*	-	-	0.0424	↑	-	-	-	-
CD16dim CD62L+\ CD11b+ (%)	-	-	-	-	-	-	-	-	-	-
CD16dim CD62L+\ CD11b+ (MFI)	**	-	-	**	0.0002	↓	-	-	-	-
CD16dim CD62L+\ CD32+ (%)	-	*	*	-	0.0208	↑	-	-	-	-
CD16dim CD62L+\ CD32+ (MFI)	-	-	***	-	0.0048	↑	-	***	0.0004	↑
Monocytes										
**All monocytes (%)**	-	-	-	-	-	-	-	-	-	-
CD11b+ (%)	-	-	-	-	-	-	-	-	-	-
CD11b+ (MFI)	-	-	-	-	-	-	-	-	-	-
CD62L+ (%)	-	-	-	-	-	-	-	-	-	-
CD62L+ (MFI)	-	-	-	-	-	-	-	-	-	-
CD32+ (%)	**	-	*	**	0.0034	↑	-	-	-	-
CD32+ (MFI)	-	-	**	*	0.0151	↑	-	*	0.0411	↑
HLA-DR+ (%)	-	-	-	-	-	-	***	***	< 0.0001	↓
HLA-DR+ (MFI)	***	***	***	***	< 0.0001	↓	***	***	< 0.0001	↓
**classical_ CD14++ CD16- (%)**	-	-	-	-	-	-	-	-	-	-
CD14++CD16-\CD11b+ (%)	-	-	-	-	-	-	-	-	-	-
CD14++CD16-\ CD11b+ (MFI)	-	-	-	-	-	-	-	-	-	-
CD14++CD16-\ CD32+ (%)	*	-	*	-	0.0379	↑	-	-	-	-
CD14++CD16- \ CD32+ (MFI)	-	-	-	-	-	-	-	***	0.0009	↑
CD14++CD16-\ CD62L+ (%)	-	*	**	*	0.005	↑	-	-	-	-
CD14++CD16- \ CD62L+ (MFI)	-	-	-	-	-	-	-	-	-	-
CD14++CD16-\ HLA-DR+ (%)	-	*	***	***	< 0.0001	↓	-	-	-	-
CD14++CD16- \ HLA-DR+ (MFI)	**	***	***	***	< 0.0001	↓	***	***	< 0.0001	↓
**anti-inflammatory_CD14++CD16+ (%)**	-	-	-	-	-	-	-	-	-	-
CD14++CD16+\ CD11b+ (%)	-	-	-	-	-	-	-	-	-	-
CD14++CD16+\CD11b+ (MFI)	-	-	-	-	-	-	-	-	-	-
CD14++CD16+\ CD32+ (%)	-	-	-	-	-	-	-	-	-	-
CD14++CD16+\ CD32+ (MFI)	-	-	***	*	0.0016	↑	-	*	0.0125	↑
CD14++CD16+\ CD62L+ (%)	*	**	**	-	0.0056	↑	-	-	-	-
CD14++CD16+\ CD62L+ (MFI)	-	-	-	-	-	-	-	-	-	-
CD14++CD16+\ HLA-DR+ (%)	-	-	-	*	0.0448	↓	-	-	-	-
CD14++CD16+\ HLA-DR+ (MFI)	-	*	-	-	0.0464	↓	-	-	-	-
**pro_inflammatory_CD14dim CD16+ (%**	-	-	-	-	-	-	-	-	-	-
CD14dim CD16+\CD11b+ (%)	-	-	-	-	-	-	-	-	-	-
CD14dim CD16+ \ CD11b+ (MFI)	-	-	-	-	-	-	-	-	-	-
CD14dim CD16+\ CD32+ (%)	*	-	-	**	0.011	↑	-	-	-	-
CD14dim CD16+ \ CD32+ (MFI)	-	-	**	*	0.0025	↑	-	*	0.0002	↑
CD14dim CD16+\ CD62L+ (%)	-	-	-	-	-	-	-	-	-	-
CD14dim CD16+ \ CD62L+ (MFI)	-	-	-	-	.-	-	-	-	-	-
CD14dim CD16+\ HLA-DR+ (%)	-	-	-	-	-	-	-	-	-	-
CD14dim CD16+ \ HLA-DR+ (MFI)	-	**	**	*	0.0063	↓	-	-	-	-

[1] Vogelgesang et al. 2008
