# Supplementary figures and images for: Immune Alterations Following Neurological Disorders: A Comparison of Stroke and Seizures
Source: Front Neurol. 2020 Jun 2;11:425. doi: 10.3389/fneur.2020.00425 (PMC7280464; doi:10.3389/fneur.2020.00425)

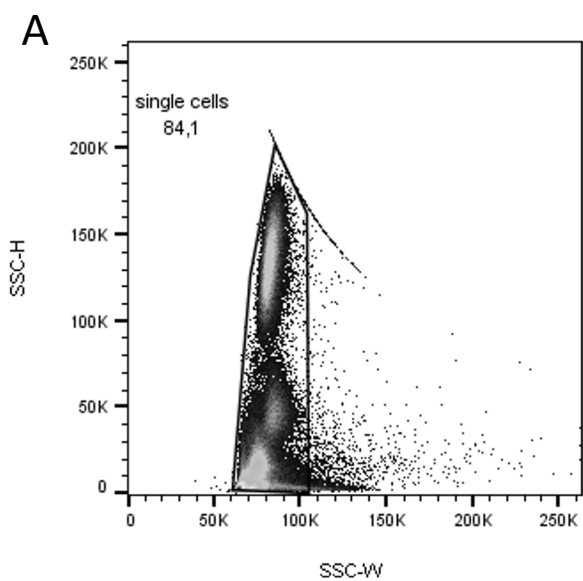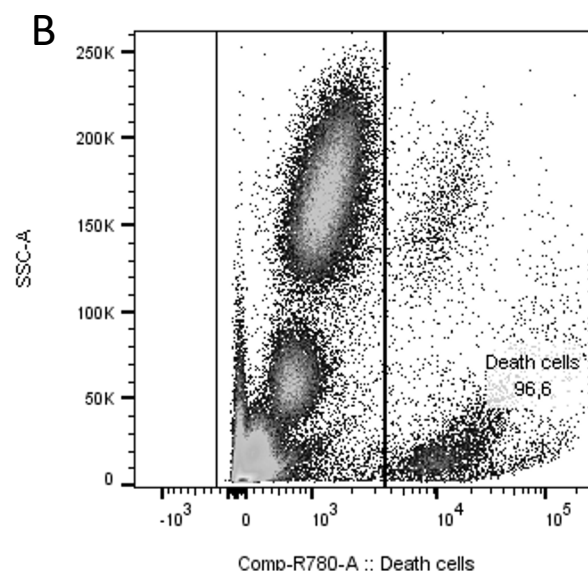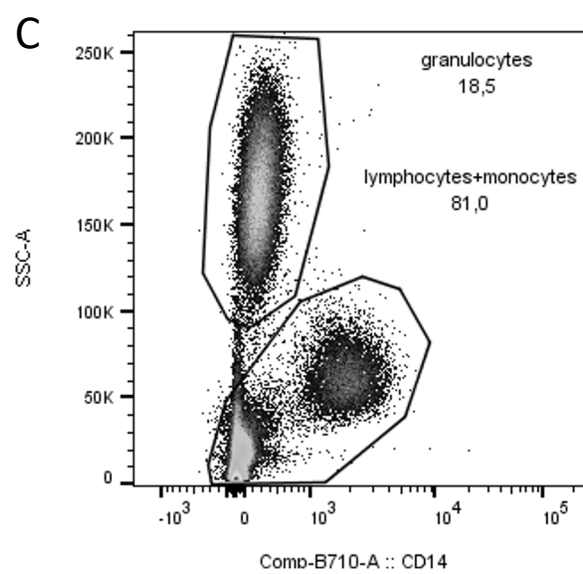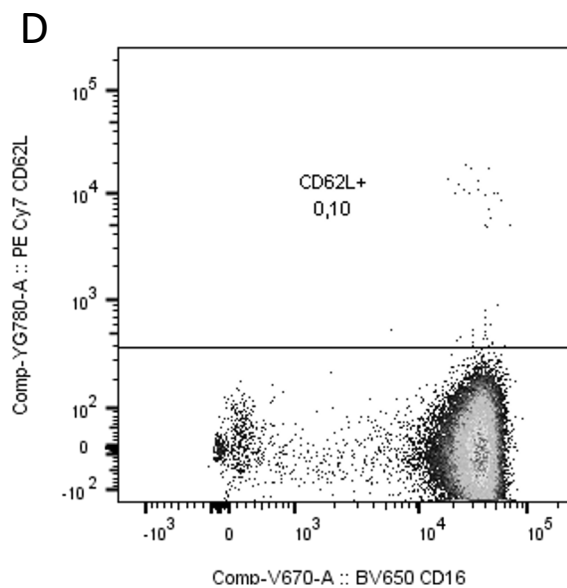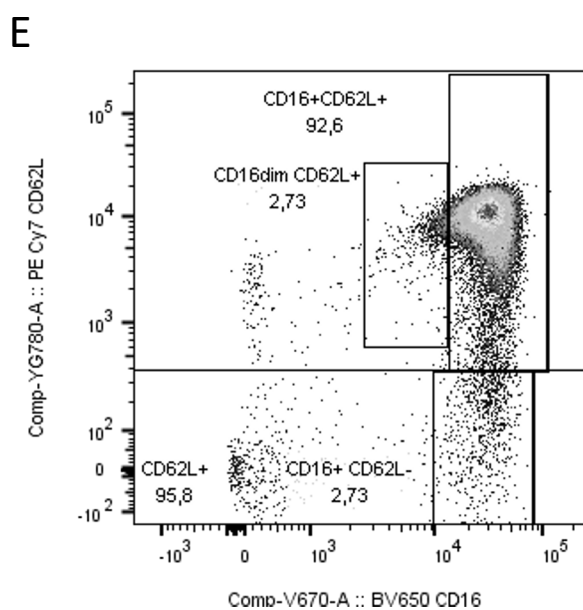

Supplement: Supplementary Figure 1A — Gating strategy for granulocyte subpopulations. Representative probe for activation marker on granulocyte subpopulation. After single cell gating (A) and determination of living cells by ZOMBIE (B), cells were gated by SSC-A (C) and subpopulation marker CD62L in their subpopulation according to FMO (D). CD16dim neutrophil population (E) was distinguished by gating the 25th percentile of main neutrophil population. [file Data_Sheet_4.PDF]

**A**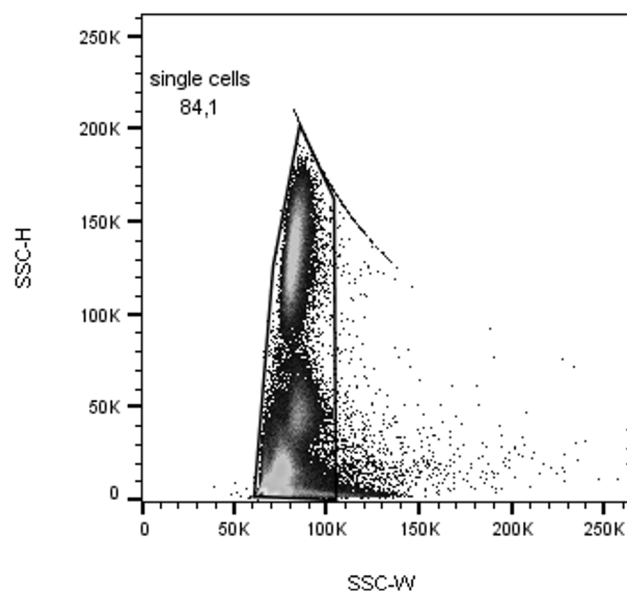**B**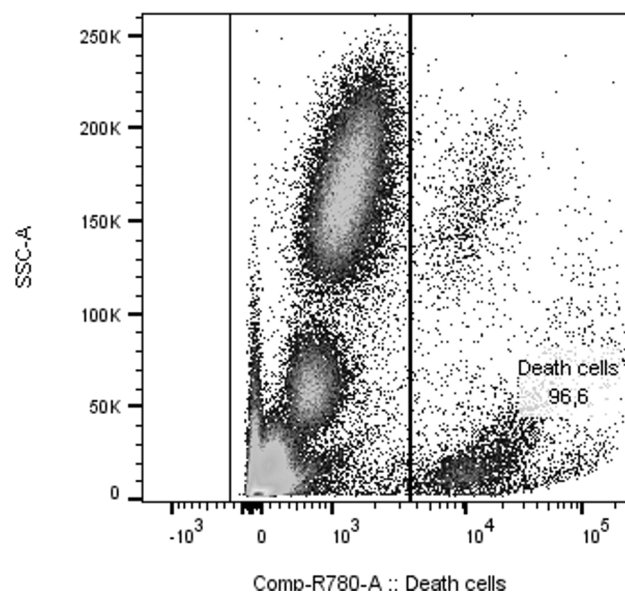**C**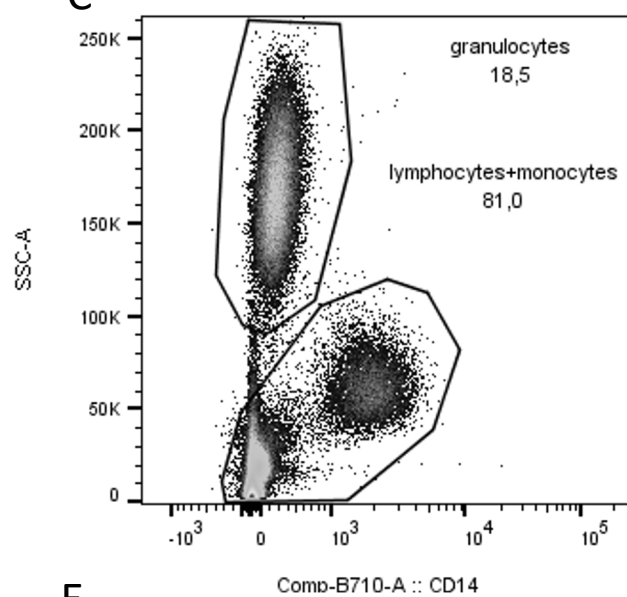**D**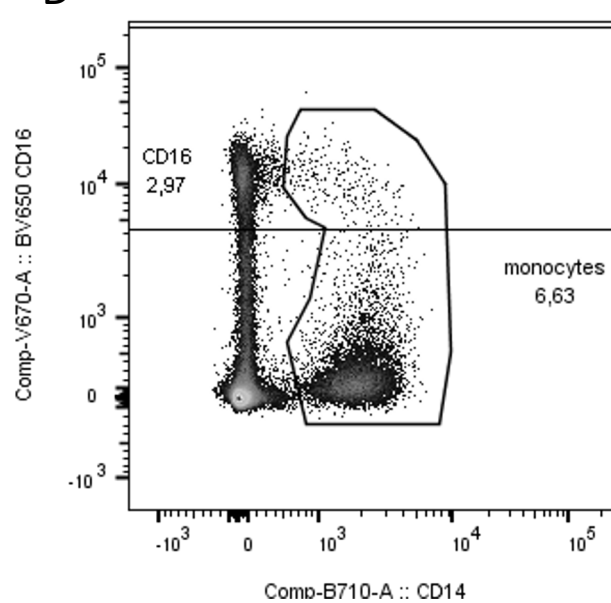**E**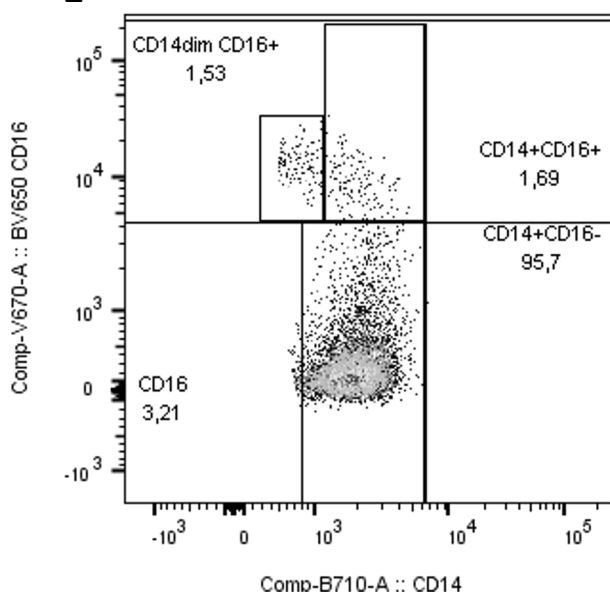

Supplement: Supplementary Figure 1B — Gating strategy for monocyte subpopulations: Representative probe for activation marker on granulocyte subpopulation. After single cell gating (A) and determination of living cells by ZOMBIE (B), cells were gated by SSC-A (C) and subpopulation marker (CD14) in their subpopulation according to FMO (D). CD14dim monocytes (E) population was distinguished by gating the 25th percentile of main neutrophil population. [file Data_Sheet_5.PDF]
